# Further evidence of clinical benefit associated with participation in phase I oncology trials

**DOI:** 10.1038/sj.bjc.6604206

**Published:** 2008-03-18

**Authors:** M Markman

**Affiliations:** 1 University of Texas M.D. Anderson Cancer Center, 1515 Holcombe Boulevard, Mail Box 243, Houston, TX 77030, USA

The issue of the legitimate therapeutic potential associated with patient participation as research subjects in phase 1 antineoplastic clinical trials has generated considerable, and often quite contentious, debate.

Some have strongly argued the chances that clinical benefit will be attained by patients entered into these studies are so remote that individuals who believe, and physicians who suggest, such an outcome is a realistic goal are suffering from what has been described as a therapeutic misconception (Appelbaum *et al*, 1987; Miller, 2000). Furthermore, it is apparently felt by a group of these commentators that major efforts are required to be certain that this ‘misconception’ is dispelled before a cancer patient is permitted to enter a phase 1 oncology trial. In support of their contention, several authors have quoted older literature that revealed that objective response rates of <5–6% are associated with phase 1 antineoplastic drug studies (Estey *et al*, 1986; Decoster *et al*, 1990; Von Hoff and Turner, 1991; Itoh *et al*, 1994). In addition, it has been noted that serious toxic effects were not rare events in these early experiences in cancer drug development.

However, more recent data published in the peer-reviewed literature provide a far different picture regarding the potential for clinical benefit associated with current phase 1 trials in the oncology arena. For example, Horstmann *et al* (2005) analysed the outcome of almost 12 000 individuals who participated in 460 phase 1 trials supported by the National Cancer Institute (Bethesda, MD, USA). Overall, 10.6% of patients experienced an objective response, which increased to 17.8% when one drug included in the phase 1 regimen had previously been approved for some clinical indication by the United States Food and Drug Administration. These results are quite comparable to that anticipated for many current programmes routinely utilised as ‘standard-of-care’ in the treatment of advanced and metastatic malignancies.

Furthermore, it is critical to acknowledge that the potential for achieving truly substantial clinical benefit through participation in a phase 1 oncology study, while certainly not a common outcome at present, is also not beyond quite realistic hope. Note, for example, that 53 out of 54 chronic myelogenous leukaemia patients treated on a phase 1 trial of imatinib mesylate achieved a complete haematologic response (Druker *et al*, 2001), and that 75% of the women with advanced ovarian cancer who participated in a phase 1 trial of paclitaxel plus carboplatin attained a major objective response to this (at that time) novel combination chemotherapy regimen (Bookman *et al*, 1996).

In this issue of the *British Journal of Cancer*, Arkenau *et al* (2008), while describing an 18 months long experience with phase 1 trials at the Royal Marsden Hospital (29 studies involving 212 patients), have added highly relevant data that provide important support to the argument that patient participation as research subjects in phase 1 oncology trials is associated with the reasonable potential for clinical benefit.

Overall, an objective response rate of 9% was noted in this report, but when patients with ‘stable disease’ were included within a rationally defined category of ‘clinical benefit’, more than 50% of individuals were considered to fall within this grouping 2 months after initiation of their phase 1 treatment programme (the first time assessment of tumour status was undertaken). Of considerable interest, even at 6 months follow-up, is that one-quarter of the entire population maintained this clinical state. Treatment-related deaths were rare (0.47%), and less than 15% of patients entered into these trials had therapy discontinued secondary to excessive toxicity.

As noted by the investigators, defining the ultimate utility of these specific novel strategies will require the conduct of phase 3 randomised trials. However, the critical point made in this paper is that there was nothing in the objective outcome data of these early-phase studies that even remotely suggests that patients who agree to participate as research subjects do not have the realistic potential to achieve a genuinely meaningful degree of individual clinical benefit.

It is also reasonable to speculate, and even anticipate, that as our understanding of the complex molecular biology of cancer improves, it will be possible to ‘target’ treatment to particular patient populations with a high pretreatment probability of achieving both an objective response and more subjective clinical benefit from the specific treatment programme (Druker *et al*, 2001). In addition, as noted by Arkenau *et al* (2008), the establishment, and ultimate routine use, of more objective metrics to select patients for participation in phase 1 trials may result in a more favourable risk-to-benefit ratio associated with research subject participation in this group of early-stage cancer drug development studies.

Based on increasingly solid data, it is reasonable to conclude that future proclamations of the fundamental absence of therapeutic intent associated with phase 1 antineoplastic drug trials simply do not reflect the current reality of cancer drug development. However, it is also important to acknowledge that there are particular phase 1 oncology trials that may appropriately be considered to be purely investigative in intent (eg, study examining a single very low dose of a cytostatic agent attached to a novel isotope designed to image distribution of the delivered material).

Finally, it is this commentator's hope that future discussions of the legitimate potential for clinical benefit associated with participation in early-stage cancer trials will increasingly focus on the specifics of the study (eg, agents to be employed; prior knowledge of activity; strength of preclinical data suggesting utility, etc.) rather than on the simple description of the ‘phase’ of the trial. It is relevant to note that agreement by clinical investigators, treating oncologists, various regulatory agencies, and institutional ethical review boards, regarding this critical issue may ultimately result in an improvement in the process of obtaining meaningful informed consent from individuals being considered for entry into these important studies.
